# Examining resilience, moral sensitivity, and work engagement among Chinese anesthesia nurses

**DOI:** 10.3389/fpsyg.2026.1874025

**Published:** 2026-07-28

**Authors:** Guoting Ma, Lingkai Wang, Qian Yang, Jing Li

**Affiliations:** 1 Department of Nursing, Shanghai Sixth People’s Hospital Affiliated to Shanghai Jiao Tong University School of Medicine, Shanghai, China; 2 Department of Intensive Care Unit, Gansu Provincial Hospital, Lanzhou, China; 3 School of Nursing, Chengdu Medical College, Chengdu, China

**Keywords:** anesthesia nurses, moral sensitivity, resilience, task shifting, work engagement

## Abstract

**Background:**

Facing rising global surgical volumes and healthcare workforce shortages, task shifting in anesthesia care has been widely implemented, including in China where a specialized anesthesia nursing workforce has rapidly expanded to approximately 60,000. However, this workforce confronts unique challenges including historical professional restrictions and the absence of standardized specialized education, making their work engagement a critical yet underexplored issue.

**Aims:**

The purpose of this study is to explore whether and how resilience may relate to work engagement among Chinese anesthesia nurses, with a particular focus on the potential mediating association of moral sensitivity in this relationship.

**Design:**

This study is a cross-sectional study.

**Methods:**

A total of 372 Chinese anesthesia nurses from 41 hospitals were surveyed using an online questionnaire that included nurses’ sociodemographic characteristics, resilience, moral sensitivity, and work engagement. The mediating effect was analyzed using Model 4 of the PROCESS macro for SPSS 27.0.

**Results:**

The total score of work engagement among Chinese anesthesia nurses was 31.25 ± 7.75. The results showed positive correlations between resilience, moral sensitivity, and work engagement (all *p* < 0.01). The indirect effect of resilience on work engagement through moral sensitivity was statistically significant (effect = 0.338), accounting for 53.7% of the total effect.

**Conclusion:**

Chinese anesthesia nurses present a relatively lower level of work engagement compared to previous reports in similar nursing populations. The results are statistically consistent with a mediating association in which moral sensitivity links resilience to work engagement among Chinese anesthesia nurses.

## Introduction

In response to the dual challenges of continuously growing global surgical volumes and the healthcare workforce crisis, many countries have implemented task shifting strategies (Federspiel et al., 2015; Bognini et al., 2023). Task shifting refers to the transfer of specific clinical responsibilities traditionally undertaken by specialist physicians to associate clinicians or non-specialist physicians (Joshi et al., 2018). This approach is applicable to countries across all geographic regions and income groups (Federspiel et al., 2015). A global review and survey revealed that 30 countries have adopted surgical task shifting, while 108 countries rely on anesthesia task shifting (Federspiel et al., 2015). One pivotal aspect of anesthesia task shifting involves the delegation of specific clinical duties, including but not limited to preoperative anesthesia assessment and postoperative recovery management, from anesthesiologists to anesthesia nurses (Henschke et al., 2025; Yin et al., 2025; Kristoffersen et al., 2022). In this context, the National Health Commission of the People’s Republic of China issued a policy in 2017 that explicitly proposed the establishment of anesthesia care units and the training of anesthesia nurses to alleviate human resource burdens and enhance the quality of anesthesia care. This policy marked the beginning of a new phase of rapid development in the construction of China’s anesthesia nursing system.

According to the latest data released during China Anesthesia Week, the total number of anesthesia nurses in China has reached approximately 60,000 in 2025, establishing the world’s largest anesthesia nursing workforce. However, this group continues to face unique developmental challenges. Historically, the Chinese Ministry of Health prohibited nurses from administering anesthesia beginning in 1989, which stagnated professional development for years. More critically, standardized anesthesia nursing education criteria remain underdeveloped, with most anesthesia nurses acquiring specialized skills primarily through hospital-based on-the-job training (Yin et al., 2025). These distinctive challenges make work engagement among Chinese anesthesia nurses a critical issue that requires immediate clarification, as current levels and associated factors remain inadequately understood.

Work engagement is conceptualized as a positive and fulfilling psychological state, characterizing person-job fit (Schaufeli et al., 2002). This state is constituted by three dimensions: vigor, characterized by high energy levels and mental resilience while working, and the persistence to overcome difficulties; dedication, referring to being strongly involved in one’s work and experiencing a sense of significance, enthusiasm, inspiration, pride, and challenge; and absorption, which is characterized by being fully concentrated and immersed in one’s work, whereby time passes quickly and one has difficulty detaching from it (Schaufeli et al., 2006). In recent years, a growing body of research has been devoted to nurses’ work engagement. Studies have confirmed that work engagement is associated with nurse perception of unit care quality (Parr et al., 2021; Dong et al., 2020). It not only relates to patient health outcomes (Tang et al., 2022; Wee and Lai, 2022) but also is strongly associated with self-reported job outcomes among nurses (Keyko et al., 2016; Van Bogaert et al., 2017). Additionally, evidence indicates that the positive outcomes of work engagement serve as a valuable buffer against the negative impact of depression on nurses’ life satisfaction (Morales-García et al., 2024). Conversely, low levels of work engagement make individuals more prone to disengagement and job burnout, ultimately contributing to turnover intention among nurses (Saito et al., 2018; Poku et al., 2025). Therefore, enhancing work engagement among anesthesia nurses is crucial for achieving positive organizational outcomes, and a potential first step is to identify modifiable factors and their underlying mechanisms.

Among these factors, resilience has garnered increasing attention. Resilience originally referred to an individual’s capacity to positively adapt to difficult conditions and challenges (Werner and Smith, 1982). As the concept has evolved, its connotation has expanded from personal traits to the interactive process between individuals and their environment. Ungar (2014) further defined resilience as the capacity of individuals (on their own or collectively) to navigate toward the culturally relevant resources they need to thrive in the face of adversity, as well as their capacity to negotiate for these resources to be provided in meaningful ways. In the field of nursing, resilience is more specifically conceptualized as a nurse’s personal self-efficacy, inherent drive to cope, and resulting ability to confidently handle difficult situations (Stacey and Cook, 2019). As a core component of positive psychological capital, resilience has been incorporated into the theoretical category of job resources (Kuhlmann and Süß, 2024). The conservation of resources theory provides a robust theoretical framework for understanding the association between resilience and work engagement (Alvaro et al., 2010). This theory posits that individuals have a fundamental motivation to acquire, retain, and protect their psychological resources. From this perspective, resilience may serve as a key personal resource that helps individuals resist resource depletion in the work environment and build resource gain cycles, which may in turn be associated with sustained work engagement (Chen et al., 2024; van Woerkom et al., 2016). Multiple studies have reported that higher resilience is associated with lower levels of compassion fatigue and depersonalization among nurses (Wei et al., 2025; Nantsupawat et al., 2024), stronger professional identity (Mousazadeh et al., 2025a), and greater intrinsic motivation to provide high-quality care to patients, which may in turn be linked to higher levels of work engagement (Liu et al., 2024; Meng et al., 2023). However, this relationship remains uncertain among Chinese anesthesia nurses, who are experiencing rapid professional role evolution and facing structural challenges. Therefore, it is particularly necessary to systematically examine the association between resilience and work engagement among Chinese anesthesia nurses.

In nursing practice, moral distress represents a prevalent and persistent problem for today’s nurses and specialist nurses in surgical care (Demir et al., 2024). Studies show that moral distress is associated with a range of negative outcomes among nurses, including physical and mental exhaustion (Boone et al., 2023), increased instances of missed care (Taskiran Eskici et al., 2025), and even turnover intention (Maben et al., 2022). Within this context, moral sensitivity is recognized as a crucial factor in understanding and managing moral distress (Kovanci and Atli, 2024). Moral sensitivity is defined as a personal trait that enables individuals to identify moral conflicts, empathize with vulnerable populations, and recognize the ethical implications of decisions made for others (Ohnishi et al., 2019). According to Rest’s Four-Component Model framework, moral sensitivity serves as an essential prerequisite for moral decision-making (Rest et al., 1999). Moral behavior begins with moral sensitivity, followed sequentially by moral judgment, moral motivation, and ultimately moral action. Nurses’ moral sensitivity is demonstrated by making ethical decisions with wisdom and compassion, while attending to patients’ physical, emotional, and spiritual needs and navigating the uncertainties of nursing practice (Mohammadi et al., 2022). Nurses with higher moral sensitivity demonstrate more positive attitudes toward patient safety and elderly caring (Kim et al., 2022). This enables them to develop more personalized care plans in complex clinical situations, while providing higher levels of spiritual care (Li et al., 2025) and person-centered care practice (Jang et al., 2022). Therefore, we speculated that a potential link may exist between moral sensitivity and work engagement among Chinese anesthesia nurses.

The broaden-and-build theory of positive emotions posits that positive emotional experiences broaden individuals’ knowledge and action capability, and build enduring personal resources (e.g., social connections, optimism, or resilience) (Fredrickson, 1998). This process creates an “upward spiral” wherein these resources and their resulting positive outcomes (e.g., satisfaction with life, flourishing and mental health) further generate positive emotions, thereby facilitating continuous personal growth and development (Fredrickson, 2004). Within this theoretical framework, resilience may help anesthesia nurses maintain positive emotional states, providing them with psychological capital to cope with professional challenges, which may in turn facilitate the functional expression of moral sensitivity (Zhai et al., 2025). When confronting ethical dilemmas, this resource support may transform moral sensitivity from mere stress perception into constructive ethical insight, which may consequently relate to nurses’ sense of meaning and accomplishment at work and ultimately be associated with their work engagement (Ahansaz et al., 2024). However, empirical research examining the relationships among these three constructs remains scarce, particularly within the Chinese cultural context.

Taken together, the above theoretical frameworks converge to support the proposed sequential process from resilience to work engagement via moral sensitivity. Conservation of Resources theory positions resilience as a key personal resource that contributes to sustained psychological capital, Rest’s model identifies moral sensitivity as the foundational step for ethical behavior, and the Broaden-and-Build theory suggests that positive emotions associated with resilience broaden cognitive resources, thereby fostering ethical awareness and, subsequently, work engagement. Therefore, moral sensitivity is hypothesized to mediate the association between resilience and work engagement.

Based on the above content, this study proposes the following research hypotheses: Hypothesis 1: resilience is positively correlated with work engagement; Hypothesis 2: moral sensitivity is positively correlated with work engagement; Hypothesis 3: resilience is positively correlated with moral sensitivity; and Hypothesis 4: moral sensitivity exhibits a mediating association between resilience and work engagement.

## Materials and methods

### Design, setting, and participants

In this multicenter cross-sectional study, we used convenience sampling to recruit anesthesia nurses from 41 tertiary hospitals across 10 provincial-level regions in China for questionnaire surveys between September and December 2025. The inclusion criteria were as follows: (i) registered nurses who had worked in the anesthesia departments for 1 year or more; (ii) nurses who gave informed consent and voluntarily participated in this study. Exclusion criteria included: (i) nurses who were not on duty during the survey period; (ii) rotating nurses or internship nursing students; (iii) those who withdrew from the study.

The sample size was estimated using G-power 3.1 software. The parameters were set as follows: *α* = 0.05, effect size (*f*^2^) = 0.10, power (1 – *β*) = 0.95, and the number of predictors (*k*) = 8, and this yielded a minimum required sample size of 172 participants. To account for an anticipated 20% questionnaire response attrition rate and include a 15% margin of safety for the convenience sampling method, the theoretical sample size was adjusted to 248.

### Instruments

The sociodemographic characteristics and occupational data questionnaire was developed by researchers through a review of the literature. This questionnaire includes 9 items, such as gender, age, education level, marital status, having children, professional title, income, working experience in anesthesiology, and received anesthesiology specialist nurse training from the Chinese Nursing Association.

Sinclair and Wallston (2004) developed the Brief Resilient Coping Scale (BRCS) to assess nurses’ tendencies to cope with stress in a highly adaptive manner. The scale contains 4 items: “I look for creative ways to alter difficult situations,” “Regardless of what happens to me, I believe I can control my reaction to it,” “I believe I can grow in positive ways by dealing with difficult situations,” and “I actively look for ways to replace the losses I encounter in life.” Responses are scored using a 5-point Likert scale, with options ranging from “*strongly disagree*” (1 point) to “*strongly agree*” (5 points). The total score ranges from 4 to 20. A score of 4–13 indicates low resilience, 14–16 indicates moderate resilience, and 17–20 indicates high resilience (Serafin et al., 2025). In this study, the Cronbach’s alpha reliability coefficient for the BRCS was 0.878.

Lützén et al. (2006) developed the Moral Sensitivity Questionnaire (MSQ) in 2006. Huang et al. (2016) translated it into Chinese and revised the original three-factor model into a two-factor model, resulting in the Moral Sensitivity Questionnaire-Revised Version into Chinese (MSQ-R-CV). The MSQ-R-CV scale was used to assess nurses’ moral sensitivity. The questionnaire comprises 9 items across two dimensions: moral responsibility and strength (5 items) and moral burden (4 items). Each item is rated on a 6-point Likert scale ranging from 1 (*strongly disagree*) to 6 (*strongly agree*). The total score ranges from 9 to 54, with higher scores indicating greater moral sensitivity. In this study, the Cronbach’s alpha coefficient for the scale was found to be 0.921.

The Utrecht Work Engagement 9-item Scale (UWES-9) was used to evaluate the work engagement levels of participants. This scale was revised by Schaufeli et al. (2006) from the original 17-item version, with the Chinese version translated by Li et al. (2013). The UWES-9 comprises three dimensions: vigor (3 items), dedication (3 items), and absorption (3 items). Each item is rated on a 7-point Likert scale, ranging from “*never*” to “*always*,” with scores assigned from 0 to 6. The total score ranges from 0 to 54 points, where a higher score indicates a greater work engagement state. In this study, the scale demonstrated a Cronbach’s alpha reliability coefficient of 0.896.

### Data collection

With the assistance of the nursing department directors or head nurses of the anesthesiology departments at our collaborating hospitals, we conducted this survey. Prior to the investigation, all research personnel received standardized training. They screened potential participants strictly according to the inclusion and exclusion criteria and subsequently sent to the eligible nurses a link to an anonymous online questionnaire via WeChat, a widely used social platform in China.

The questionnaire was structured as follows: The first page of the electronic questionnaire served as the informed consent form, which clearly explained the study’s purpose, procedures, estimated completion time, confidentiality measures, and participants’ rights. It explicitly stated that participants who proceeded to complete and submit the subsequent anonymous questionnaire would be deemed to have agreed to participate in the study. Upon completing this section, the survey questions were automatically presented.

To ensure data accuracy and validity, the system was configured to allow only one submission per device and to prevent the submission of questionnaires with incomplete mandatory fields. Following data collection, two researchers independently reviewed all entries. Questionnaires completed in less than 5 min or longer than 30 min were excluded, as were those exhibiting patterned responses (e.g., selecting the same answer for 10 or more consecutive questions).

A total of 416 questionnaires were submitted. After excluding 44 questionnaires due to abnormal completion times or patterned responses, 372 valid questionnaires were retained for the final analysis, yielding a valid questionnaire rate of 89.4%. Because the questionnaire was distributed anonymously online, instances of refusal could not be systematically recorded, and incomplete questionnaires were intercepted by the platform at the system level without entering the final data collection.

### Data analysis

Data analysis was performed using IBM SPSS 27.0. Continuous data were described as means (standard deviations), while categorical data were presented as frequency (percentage). The bivariate relationships among resilience, moral sensitivity, and work engagement were examined using Pearson correlation analysis. The mediating association of moral sensitivity between resilience and work engagement was tested using the SPSS PROCESS macro, Model 4, with the bias-corrected bootstrap method based on 5,000 resamples and a 95% confidence interval (CI). Covariates included gender (0 = female, 1 = male), age (1 = 18–25, 2 = 26–30, 3 = 31–35, 4 = 36–40, 5 = 41–45, 6 = above 45 years), education level (1 = junior college and less, 2 = undergraduate, 3 = postgraduate and more), marital status (1 = single, 2 = married, 3 = divorce or other), having children (0 = no, 1 = yes), professional title (1 = nurse, 2 = the primary nurse practitioner, 3 = nurse-in-charge, 4 = deputy director nurse or above), income (1 = <3,000, 2 = 3,000–5,000, 3 = 5,001–10,000, 4 = > 10,000 RMB/month), working experience in anesthesiology department (1 = <3, 2 = 3–5, 3 = 6–10, 4 = 11–15, 5 = > 15 years), and received anesthesiology specialist nurse training from the Chinese Nursing Association (0 = no, 1 = yes). Among these covariates, binary variables (gender, having children, specialist nurse training) were entered as 0/1 indicators. Ordinal categorical variables (age, education level, professional title, income, working experience) were entered as continuous variables based on their inherent ordering. For the nominal variable marital status, two dummy variables were created with “single” as the reference category, and these dummy variables were entered into the PROCESS model. If the 95% CI did not contain zero, the effect was considered statistically significant. To assess the potential risk of common method bias, Harman’s single-factor test was conducted by entering all 22 items from the BRCS, MSQ-R-CV, and UWES-9 into an unrotated exploratory factor analysis.

### Ethics approval

This study was conducted in strict accordance with the ethical principles of the Declaration of Helsinki and was approved by the Ethics Committee of Gansu Provincial Hospital (approval number: No. 2025–538). Participants provided electronic written informed consent (by clicking to proceed after reading the consent information on the first page of the questionnaire). Participants reserved the right to withdraw from the study at any time. Regarding data management, the research employed anonymous collection and de-identification processes. All data were accessible only to authorized research team members under confidential conditions and were used exclusively for statistical analysis in this study.

## Results

### Participant characteristics

Of the 372 anesthesia nurses included in the final analysis, the majority were women (84.9%), and the mean age was 33.53 ± 5.72. Notably, over one-third of the participants had fewer than 6 years of work experience in anesthesiology (37.4%), and only 29.8% had received anesthesiology specialist nurse training from the Chinese Nursing Association. Additional information is detailed in Table 1.

**Table 1:** Participant characteristics (*n* = 372).

Variables	Mean (SD)/*n* (%)
Gender	
Female	316 (84.9)
Male	56 (15.1)
Age (years)	
18–25	23 (6.2)
26–30	94 (25.3)
31–35	133 (35.8)
36–40	81 (21.8)
41–45	29 (7.8)
Above 45	12 (3.2)
Education level	
Junior college and less	17 (4.6)
Undergraduate	329 (88.4)
Postgraduate and more	26 (7.0)
Marital status	
Single	80 (21.5)
Married	286 (76.9)
Divorce or other	6 (1.6)
Having children	
No	109 (29.3)
Yes	263 (70.7)
Professional title	
Nurse	53 (14.2)
The primary nurse practitioner	97 (26.1)
Nurse-in-charge	198 (53.2)
Deputy director nurse or above	24 (6.5)
Income (RMB/month)	
<3,000	25 (6.7)
3,000–5,000	103 (27.7)
5,001–10,000	168 (45.2)
>10,000	76 (20.4)
Working experience in anesthesiology (years)	
<3	84 (22.6)
3–5	55 (14.8)
6–10	109 (29.3)
11–15	86 (23.1)
>15	38 (10.2)
Received anesthesiology specialist nurse training from the Chinese Nursing Association	
No	261 (70.2)
Yes	111 (29.8)

SD, standard deviation; RMB, renminbi.

### Descriptive statistics and variable correlation analysis

Harman’s single-factor test showed that the first unrotated factor accounted for 48.987% of the total variance. In this study, the mean scores for resilience, moral sensitivity, and work engagement among anesthesia nurses were 12.33 ± 3.20, 40.74 ± 8.50, and 31.25 ± 7.75, respectively. The scores for specific dimensions are presented in Table 2. Pearson correlation analyses showed that work engagement was positively correlated with both moral sensitivity (*r* = 0.723, *p* < 0.01) and resilience (*r* = 0.620, *p* < 0.01). Moral sensitivity was also positively correlated with resilience (*r* = 0.623, *p* < 0.01).

**Table 2:** Mean scores and correlation coefficients of resilience, moral sensitivity, and work engagement (*n* = 372).

Variables	Mean ± SD	R	M	M1	M2	W	W1	W2	W3
R	12.33 ± 3.20	1							
M	40.74 ± 8.50	0.623^**^	1						
M1	23.28 ± 5.24	0.620^**^	0.950^**^	1					
M2	17.47 ± 3.88	0.527^**^	0.907^**^	0.730^**^	1				
W	31.25 ± 7.75	0.620^**^	0.723^**^	0.703^**^	0.633^**^	1			
W1	11.81 ± 3.47	0.609^**^	0.737^**^	0.729^**^	0.629^**^	0.907^**^	1		
W2	10.38 ± 3.73	0.506^**^	0.593^**^	0.559^**^	0.544^**^	0.899^**^	0.744^**^	1	
W3	11.12 ± 3.46	0.548^**^	0.619^**^	0.612^**^	0.528^**^	0.900^**^	0.731^**^	0.765^**^	1

R, resilience; M, moral sensitivity; M1, moral responsibility and strength; M2, moral burden; W, work engagement; W1, vigor; W2, dedication; W3, absorption.

SD, standard deviation.

∗∗*p* < 0.01.

### Mediating association of moral sensitivity between resilience and work engagement

**Figure 1:**
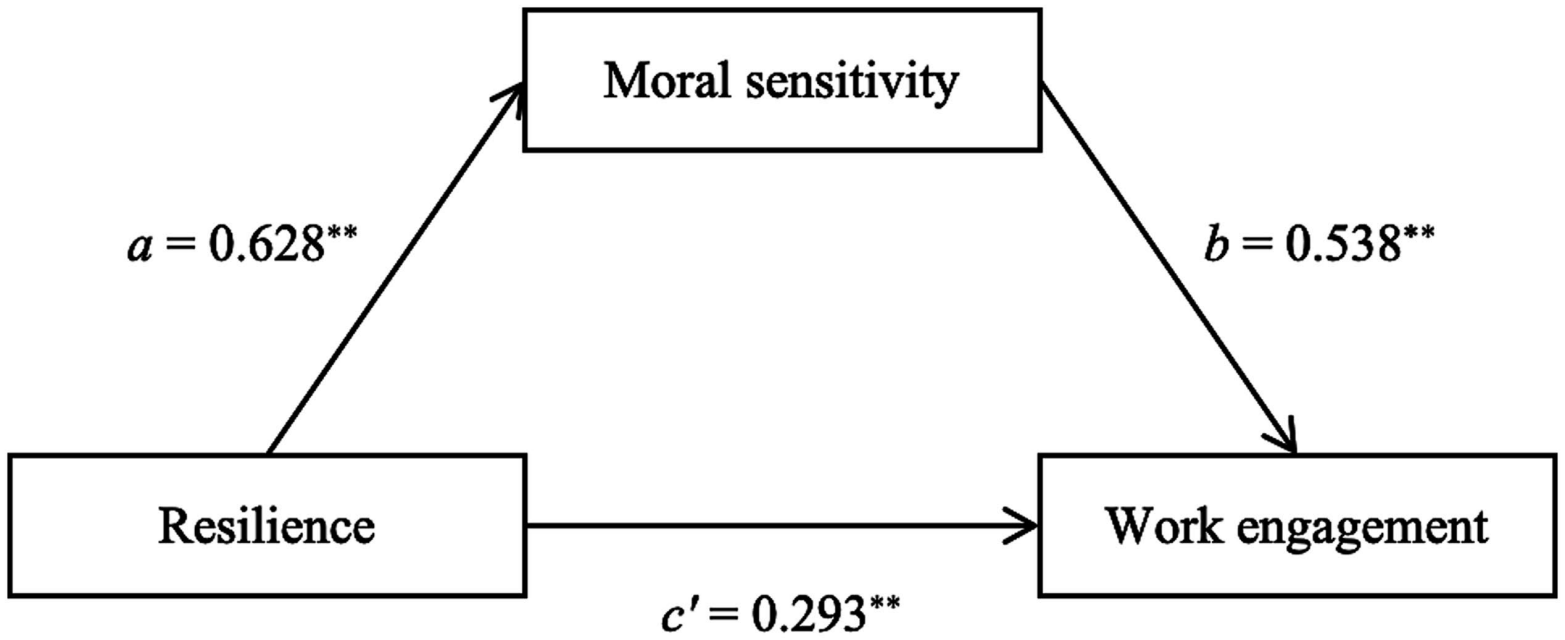
Mediation model of moral sensitivity on the association between resilience and work engagement **denotes *p* < 0.001.

This study examined the mediating association of moral sensitivity between resilience and work engagement, controlling for gender, age, education level, marital status, having children, professional title, income, working experience in anesthesiology department, and received anesthesiology specialist nurse training from the Chinese Nursing Association. The results showed a significant total effect of resilience on work engagement (*c* = 0.630, *p* < 0.001). Specifically, the direct effect of resilience on work engagement was found to be significant (*c*′ = 0.293, *p* < 0.001). Furthermore, the bias-corrected bootstrap method revealed a significant indirect effect of resilience on work engagement through moral sensitivity (effect = 0.338, Boot SE = 0.036, Boot 95% CI [0.269, 0.410]). This indirect effect accounted for 53.7% of the total effect. Detailed information is presented in Figure 1 and Table 3.

**Table 3:** Mediating association of moral sensitivity between resilience and work engagement (*n* = 372).

Effect	Path	*B*	*β*	SE	*t*	*p*	95% CI
Direct effect	Resilience ⟶ work engagement	0.893	0.293 (*c*′)	0.136	6.573	<0.001	[0.626, 1.160]
Indirect effect	Resilience ⟶ moral sensitivity	1.669	0.628 (*a*)	0.108	15.454	<0.001	[1.457, 1.881]
	Moral Sensitivity ⟶ work engagement	0.617	0.538 (*b*)	0.051	12.018	<0.001	[0.516, 0.718]
Total effect		1.923	0.630 (*c*)	0.125	15.437	<0.001	[1.678, 2.168]

B, unstandardized coefficient; β, standardized coefficient; SE, standard error; CI, confidence interval.

Indirect effect (a × b): B = 1.030, Boot SE = 0.113, Boot 95% CI [0.813, 1.259]; completely standardized indirect effect = 0.338, Boot SE = 0.036, Boot 95% CI [0.269, 0.410]. Bootstrapping based on 5,000 resamples.

Adjusting for gender, age, education level, marital status, having children, professional title, income, working experience in anesthesiology department, and received anesthesiology specialist nurse training from the Chinese Nursing Association.

## Discussion

Against the backdrop of surgical task shifting, the Chinese anesthesia nursing workforce has expanded significantly and consequently plays an increasingly vital role in safeguarding perioperative patient safety. This is the first study to examine how resilience is associated with work engagement among Chinese anesthesia nurses, and to explore the mediating association of moral sensitivity in this relationship. The results revealed significant positive correlations between resilience, moral sensitivity, and work engagement. Further analysis demonstrated that resilience has a direct association with work engagement and an indirect association through the mediating association of moral sensitivity. These findings suggest that moral sensitivity may exhibit a positive association with work engagement in the context of resilience.

Nurses’ work engagement is crucial for maintaining their physical and mental well-being, enhancing the quality of care, and stabilizing the professional workforce. In this study, the work engagement score of anesthesia nurses in our sample (31.25 ± 7.75) was lower than that reported for Chinese intensive care unit nurses (33.85 ± 8.89) working in similarly high-intensity and high-stress environments (Li et al., 2022). This finding highlights systemic issues within anesthesia nursing practice in China. Structurally, more than one-third (37.4%) of anesthesia nurses had fewer than 6 years of work experience, indicating a rapidly growing yet still maturing workforce. At the same time, a striking 70.2% had not received standardized anesthesiology specialist nurse training, directly reflecting the potentially underdeveloped systematic post-licensure training and professional support systems (Yin et al., 2025). These factors collectively may undermine their professional competence and confidence. Externally, a national survey revealed a severe lack of public understanding of anesthesiology in China, where the specialized role and contributions of anesthesia nurses fail to receive adequate social recognition and respect (Ma et al., 2024). Factors such as ambiguous scopes of practice and unclear career progression pathways may further exacerbate work-related stress and burnout (Cheng et al., 2024). Therefore, comprehensive strategies involving systematic specialized training, clear definition of scopes of practice, and enhanced psychological capital are needed to improve working conditions, thereby effectively supporting anesthesia nurses and may be associated with higher work engagement.

The results showed a positive and significant association between resilience and work engagement, which is consistent with previous studies (Liu et al., 2024; Meng et al., 2023). By verifying this association among Chinese anesthesia nurses, we have further extended its cultural applicability and professional specificity. Resilience may serve as a positive correlate of work engagement, which may lie in its role as a key psychological resource that helps nurses cope with occupational challenges. Nurses with higher levels of resilience are better able to manage emotional responses in daily work, recover more quickly from professional stress, and thus adapt more effectively to workplace demands (Rogers et al., 2024). Furthermore, resilience may contribute to maintaining optimistic thinking in nursing practice. This positive cognitive orientation may promote deeper self-reflection and self-worth recognition, which may in turn be associated with professional identity (Shi et al., 2023). Some studies have found that resilience shows a strong association with work engagement, meaning that nurses with high resilience can more effectively cope with stress, maintain a positive mindset, and avoid burnout (Cao and Chen, 2021). This inherent advantage may directly provide sustained drive for their vitality, dedication, and focus on work. In the high-risk and high-responsibility context of anesthesia nursing, the supportive association of resilience on work resources may be particularly pronounced. Resilience may allow nurses not only to adapt to changes in the work environment but also to actively explore the positive meaning of their work, which may in turn be related to their perception of the value of clinical practice and supporting professional development (Yu et al., 2019). At the same time, individuals with stronger resilience tend to have higher job satisfaction and better health status. These factors together form a virtuous cycle that encourages nurses to engage more fully in their work. It is important to clarify that the composition of nurses’ resilience is not singular; its key attributes encompass multidimensional factors such as social support, self-efficacy, work-life balance/self-care, humor, optimism, and being realistic (Cooper et al., 2020). Maintaining nurses’ resilience requires joint actions and participation from both individuals and organizations (Wang et al., 2025). Therefore, organizational measures such as providing systematic psychological capital training and establishing social support networks should be implemented to actively cultivate and sustain resilience among anesthesia nurses, which may be associated with higher work engagement.

Further analysis revealed that among Chinese anesthesia nurses, resilience showed an significant indirect effect on work engagement through moral sensitivity, with an effect size of 0.338, accounting for 53.7% of the total effect (0.630). This finding reaffirms the direct association of resilience on work engagement and highlights the mediating association of moral sensitivity, which constitutes a key contribution of this study. One possible explanation for this indirect association is that resilience may provide anesthesia nurses with the psychological resources needed to address ethical challenges, which may facilitate the transition of moral sensitivity from a passive ethical awareness toward a more active professional competency. Specifically, moral sensitivity may help nurses identify ethical dimensions in nursing practice, particularly moral dilemmas in high-risk situations (Ibrahim and Ibrahim, 2025). When confronting moral conflicts, resilience resources may support nurses to actively engage their moral sensitivity, sustain moral courage, and proceed with ethical decisions. This proactive ethical practice may allow nurses to effectively navigate ethical dimensions in complex clinical environments, safeguarding patients’ rights to autonomous decision-making (Mousazadeh et al., 2025b). Meanwhile, it may enhance the quality of care through accurate assessment of patients’ emotional states and anticipation of potential conflicts (Guo et al., 2024). However, it is worth noting that the MSQ-R-CV encompasses not only moral responsibility and strength but also a moral burden dimension. While the former dimension may facilitate work engagement through enhanced ethical awareness and professional identity, the latter may reflect the psychological cost of heightened ethical sensitivity, such as distress from perceived ethical inadequacies. Future studies should examine the differential associations of these two dimensions to fully capture the complexity of moral sensitivity in nursing practice. The indirect association observed in this study can be viewed as relevant to building moral resilience. Moral resilience may support nurses in maintaining moral integrity and adhere to ethical principles when facing moral dilemmas (Liu et al., 2025). When nurses successfully address ethical challenges with the support of resilience, each successful ethical practice cumulatively strengthens their moral resilience. Furthermore, evidence suggests that moral resilience may be positively associated with work engagement and negatively associated with turnover intention (Yi et al., 2024). Therefore, cultivating moral sensitivity in anesthesia nurses should be regarded as an important component in enhancing their professional adaptability. Through systematic ethics training and practical support, helping nurses develop professional ethical decision-making capability may represent a promising direction for supporting their work engagement and long-term career development.

Several limitations should be considered. A major methodological concern is the cross-sectional design, which can only identify correlations and thus cannot establish causal associations. It should also be noted that the proposed mediation ordering is theory-driven and statistically consistent with the observed data; however, this design cannot establish it as superior to alternative temporal orderings, as reverse or reciprocal pathways remain plausible. Longitudinal studies, cross-lagged panel designs, or intervention experiments may help disentangle temporal directionality among these variables. Another issue pertains to the single-time-point self-report measures, which may introduce common method bias. The Harman single-factor test showed that the first unrotated factor accounted for 48.987% of the total variance, exceeding the 40% threshold commonly used in the literature, suggesting that common method bias cannot be ruled out. Future studies could adopt multi-time-point and multi-source data collection to further address this concern. Additionally, the data were obtained from 41 hospitals with a nested structure, and the potential hospital-level clustering effects were not modeled. Future research should consider multilevel analytical approaches to further account for such influences. Finally, anonymous distribution prevented tracking of invitees and non-completers, limiting assessment of selection bias and representativeness. Systematic documentation of participation flow is recommended for future studies.

## Conclusion

This study highlights considerable room and necessity for improving work engagement among Chinese anesthesia nurses, and reveals significant positive correlations among resilience, moral sensitivity, and work engagement. Resilience shows a direct association with work engagement and an indirect association through moral sensitivity, suggesting a synergistic link between psychological capital and ethical competence. These findings point to the potential value of efforts to help anesthesia nurses cope with occupational stress and ethical dilemmas, thereby contributing to a resilient workforce. This has relevance for stabilizing the nursing workforce, improving clinical care quality, and ultimately supporting safer patient services within a sustainable anesthesia nursing system.

## Data Availability

The datasets presented in this article are not readily available because the data that support the findings of this study are available from the corresponding author upon reasonable request. Requests to access the datasets should be directed to kaixinmama777@163.com.
